# Lights and Shadows on Managing Immune Checkpoint Inhibitors in Oncology during the COVID-19 Era

**DOI:** 10.3390/cancers13081906

**Published:** 2021-04-15

**Authors:** Chiara Burgaletto, Oronzo Brunetti, Antonio Munafò, Renato Bernardini, Nicola Silvestris, Giuseppina Cantarella, Antonella Argentiero

**Affiliations:** 1 Department of Biomedical and Biotechnological Sciences, University of Catania School of Medicine, 95123 Catania, Italy; uni317424@studium.unict.it (C.B.); uni315775@studium.unict.it (A.M.); gcantare@unict.it (G.C.); 2 IRCCS Istituto Tumori “Giovanni Paolo II”, 70124 Bari, Italy; dr.oronzo.brunetti@tiscali.it (O.B.); n.silvestris@oncologico.bari.it (N.S.); argentieroantonella@gmail.com (A.A.); 3 Department of Biomedical Sciences and Human Oncology, University of Bari “Aldo Moro”, 70124 Bari, Italy; 4 Unit of Clinical Toxicology, Policlinico “G. Rodolico”, University of Catania School of Medicine, 95123 Catania, Italy

**Keywords:** immuno-oncologics, immune response, cancer therapy, drug safety

## Abstract

**Simple Summary:**

The potential interference at the immune response level between COVID-19 and cancer therapy raises key clinical questions and points out scientific issues that need to be promptly addressed. Among the therapeutic strategies available in oncological clinics, major concerns are raised by immunomodulatory drugs and, particularly, by immune checkpoint inhibitors (ICIs), which currently constitute a crucial drug in the management of several types of advanced and metastatic solid tumors. To date, the debate about the real impact of ICIs on the clinical outcome of COVID infection is still open. Here, we report and review the results of pertinent studies designed to evaluate the relationships between ICI treatment and COVID-19.

**Abstract:**

Since the start of the global spread of coronavirus disease (COVID-19) pandemic, cancer patients were identified as a specifically susceptible subgroup of the patient population. Several reports have shown that cancer patients have an increased risk of both contracting the infection and of experiencing a more severe disease course, with a rapidly evolving picture associated with higher mortality. The assumption of cancer patients as “COVID-19 vulnerable” has led, irretrievably, to profound changes in the decision making of oncological treatments. Potential justifications for such concerns encompass the cancer-dependent suppression of the immune response, as well as the influence of administration of systemic anticancer treatments, including chemotherapy and immunotherapy. Nevertheless, to date, it is not clear whether the use of immune checkpoint inhibitors (ICIs) in cancer patients is safe, given their modulating effects on the immune system, or that they may rather conceal detrimental consequences. Theoretically, on the one hand, ICIs may enhance the immunological control of viral infections through their immunostimulating mechanisms; on the other hand, they could contribute to the hyper-inflammatory phase of COVID-19, worsening its clinical outcomes. In this study, we report the foremost clinical observations on the safety of ICI administration in cancer patients affected by COVID-19.

## 1. Introduction

The coronavirus disease 2019 (COVID-19) pandemic has brought about a significant burden on global economy and health, resulting in a total reorganization of the healthcare system in many specialties, including medical oncology [1]. Indeed, the consequences of this pandemic for oncology are strong, resulting in the rescheduling of treatments and delayed appointments. The dilemma of whether to continue, postpone, or even terminate active cancer treatments persisted, and physicians are expected to prioritize the patients’ safety and to ensure that they receive timely treatment [2]. The disease course of individuals contracting the severe acute respiratory syndrome coronavirus 2 (SARS-CoV-2) infection is phenotypically diverse, ranging from mild symptoms to respiratory failure, cytokine release syndrome (CRS), and multi-organ failure [3]. Therefore, COVID-19 can occur in patients who are frail due to old age and to the common comorbidities related, such as diabetes mellitus, obesity, chronic lung disease, hypertension, and heart diseases. All of these pathological settings correlate with an impaired immune response to pathogens. In this scenario, these factors are associated with a higher risk of developing severe or lethal SARS-CoV-2 complications [4,5]. Notably, cancer patients, known to be more susceptible to infections, have an increased risk of running a more severe disease course, with a more rapidly evolving disease associated with higher mortality, thus requiring greater levels of intensive care [6,7]. Potential explanations include the immune suppression caused by the malignancy, particularly hematological ones, and the use of systemic anticancer treatments such as chemotherapy, immunotherapy, and radiotherapy, which can exacerbate the immunosuppressive condition [8]. In fact, cancer patients receiving active chemotherapy have a high risk of contracting infections and developing infectious complications, primarily due to the effect on the myeloproliferative cells with the subsequent development of prolonged or febrile neutropenia [9,10].

## 2. Immunological and Inflammatory Features of COVID-19 Infection

The host immune response is crucial in determining the clinical course of COVID-19, as it is critically involved in the balance between the clearance of the virus, when efficient, and the propagation of the disease, when deficient [11]. Indeed, it is well recognized that the SARS-CoV-2 infection could negatively affect the host’s antiviral immunity at an initial stage of the pathological process, largely contributing to the disease’s severity [12]. In this regard, several studies have shown that the number of natural killer (NK) cells and cytotoxic lymphocytes (CTLs), whose role is particularly relevant in the control of infections, was substantially depleted in COVID-19 patients [13]. More specifically, the total number of CTLs and NK cells is markedly reduced in severe patients rather than moderate cases, pointing out that the degree of lymphopenia and the lower lymphocyte counts are associated with a poor prognosis in COVID-19 patients [14].

Notably, the expression of CD94/NK group 2 member A (NKG2A), an inhibitory receptor, known to induce NK cells exhaustion in chronic viral infection and associated with an impaired ability to produce interferon (IFN)-γ, interleukin-2 (IL-2), and tumor necrosis factor-alpha (TNF-α), is significantly increased on the NK and T cells of SARS-CoV-2 infected patients when compared with healthy controls [15]. These data strongly suggest that the NKG2A expression could be correlated with the functional exhaustion of lymphocytes and, more generally, with a dysfunctional adaptive immune system.

Similarly, critically ill COVID-19 patients also have a significantly higher expression of immunosuppressive markers, such as programmed cell death 1 (PD-1), which deeply impairs the effective functions of T cells [12].

The pathophysiological mechanisms of lymphopenia in COVID-19 patients are still unknown; however, different data propose a direct effect of SARS-CoV-2 on T cells, probably, through apoptosis [16].

These factors, in their entirety, may account for the delayed development of a maladaptive immune response and the severity of the acute phase of the SARS-CoV-2 infection. Thus, the severity of COVID-19 is not only attributable to direct viral damage but also to immune dysregulation, resulting in an excessive inflammatory response that leads to the production of extremely high levels of inflammatory cytokines, including IFN-α, IFN-γ, IL-1β, IL-6, TNF-α; the transforming growth factor (TGF-β); and chemokines, such as CCL-2, CCL-3, CCL-5, CXCL-10, resulting in severe organ damage up to the development of CRS, when uncontrolled [17]. This lethal inflammatory state, which is the result of an immune system running wild and increasing lung permeability, determines a high immune cell infiltration and promotes a further SARS-CoV-2 invasion. This, in turn, enhances the local production of cytokines and chemokines, leadings to lung disruption and, in the worst-case scenario, acute respiratory distress syndrome (ARDS), the main death cause in COVID-19 [18].

## 3. COVID-19 and Immune Checkpoint Inhibitors: Different Considerations

The high mortality rate in frail patients is a notable feature since the onset of the COVID-19 pandemic. Above all, cancer patients are the most at risk. Thus, the possible coexistence, in the same individual, of a cancer diagnosis and a COVID-19 infection may generate a synergistic negative prognostic effect [19]. Among different therapeutic strategies available in oncological clinics, major concerns are raised by immunomodulatory drugs, which, due to their pleiotropic effect on the functional activity of the immune system, have brought a profound change in the cancer treatment landscape. In this regard, immune checkpoint inhibitors (ICIs) currently represent a first-line drug class in the management of several types of advanced or metastatic solid tumors, including melanoma, lung cancer, renal carcinoma, urothelial cancer, and hematological cancers [20]. ICIs include monoclonal antibodies directed against PD-1, programmed death ligand 1 (PD-L1), and cytotoxic lymphocyte-associated protein 4 (CTLA-4). These receptors, defined as immune checkpoints, are distinctively activated by cancer cells in the attempt to suppress T cell activation and its anti-cancer activity. The negative checkpoint blockade by ICIs removes the inhibition, driving effective antitumor response through the central and peripheral immune mechanisms [20,21]. The immune checkpoint pathway is an endogenous component of the immune system, liable for harmonizing the physiological immune response and maintaining self-tolerance.

Thus, theoretically, the PD-1/PD-L1 blockade would be able to rectify the strength and quality of the T cell activity, both CTLs, and CD4 helper T cells (Ths), enhancing the ability to efficiently counteract the viral infection and reduce organ dysfunction [22]. This represents the rationale behind the clinical trials currently registered in ClinicalTrials.gov (NCT04268537; NCT04356508; NCT04343144; NCT04413838) with the common aim of studying the hidden potential of ICI administration to restore immunocompetence, enhance T cell response, and prevent the development of severe COVID-19-related complications [23].

**Figure 1:**
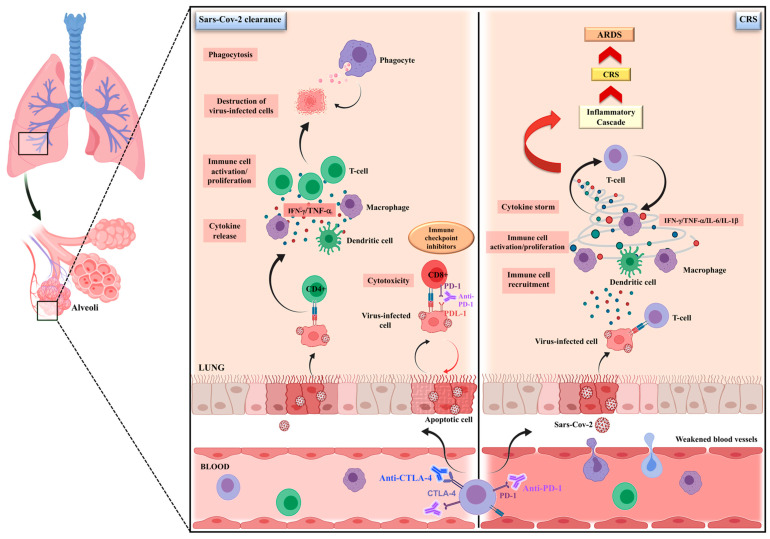
The possible dual impact of immune checkpoint inhibitors on SARS-CoV-2 infection. The debate is still open about the real impact of ICIs on SARS-CoV-2-infected cancer patients. It is well recognized that SARS-CoV-2 infection could negatively influence the host antiviral immune response, contributing to disease severity. On the one hand, by restoring the cellular immunocompetence, ICIs, can enhance the immunological control of infection, accelerating the viral clearance through an increased CD8^+^ T cell activity, as well as by an improved antigen-dependent recruitment of CD4 T cells, cytokine release and immune-cell proliferation, ultimately leading to the destruction of virus-infected cells by phagocytosis. On the other hand, ICIs may worsen the hyper-inflammatory state that, triggering the cytokine-release syndrome (CRS), leads to lung disruption and, in the worst-case scenario, an acute respiratory distress syndrome (ARDS).

Given the modulating effects on the immune system, uncertainty persists about the potential impact of the administration of ICIs to cancer patients during this pandemic. The debate is still open on the real interaction of ICIs in relation to the severity of COVID-19 [24]. Therefore, on one hand, ICIs, can enhance the immunological control of viral infection and improve the clinical outcome by restoring the cellular immunocompetence; on the other hand, this interplay may worsen the hyper-inflammation in severe cases of COVID-19 [24,25,26] (Figure 1).

In addition, patients receiving ICI may develop an interstitial pneumopathy; their clinical and radiological characteristics can overlap with COVID-19-related pneumonia [27]. Although in a meta-analysis of 26 studies, the reported incidence of ICI pneumonitis waslower than 10% [28], in clinical practice, it noticeably exceeded 19% [29]. Additionally, the time between ICI administration and the onset of checkpoint inhibitor-associated pneumonitis varied from 2 to 24 months, with a median time of 2.8 months [30]. Moreover, whilst ICI-associated pneumonitis may exacerbate pulmonary inflammation, COVID-19 infection may mask the symptoms of lungs’ immune-related adverse events, potentially postponing essential treatment [31]. The exact differential diagnosis would become crucial for providing the most appropriate treatment, and a multidisciplinary approach based on laboratory, radiographical, and pathological parameters may be helpful in discriminating between these two clinical entities [32]. Lastly, physicians should answer the challenging question of whether interactions are possible between treatments for COVID-19 and anticancer agents [33].

## 4. ICIs’ Impact on COVID-19 Outcome in Cancer Patients: Clinical Data

Since the first reports, assessing the impact of ICIs on the clinical outcome of cancer patients with SARS-CoV-2 infection, produced contrasting results, the administration of this particular pharmacological class represents an issue that requires further investigations.

A single-center, retrospective study, conducted at Memorial Sloan Kettering Cancer Center, which analyzed a heterogeneous group of 423 patients with various cancer and symptomatic COVID-19, reported that the age of over 65 years and treatment with ICIs were predictors for hospitalization and respiratory illness, regardless of cancer type and other comorbidities [34]. Along the same lines, a retrospective study examined the medical records of two Chinese tertiary cancer institutions. In particular, the authors analyzed the data of 11 cancer patients who had prior exposure to ICIs and were subsequently diagnosed with COVID-19. They observed that six out of seven patients, who developed severe COVID-19 disease, received three or more cycles of ICIs. Notably, four patients with lung cancer developed a severe clinical course. They hypothesized that patients with a long course of ICIs would more likely develop a severe phenotype of the disease. Nevertheless, this finding was not statically significant, possibly due to the small size of the study cohort [35].

Other studies have examined the role of ICI immunotherapy as a risk factor in not achieving a similar association. In particular, Luo et al., analyzing 69 patients with lung cancer, who were diagnosed with COVID-19, examined the association between COVID-19 severity and the receipt of PD-1 blockade therapy. Although it was confirmed that lung cancer patients represent a particularly vulnerable population with high rates of severe COVID-19, Luo and colleagues did not find a discernible relationship between PD-1 blockade and an increased risk of severe COVID-19 in terms of a predefined composite rate of intensive care unit (ICU), intubation, transition to do not intubate (DNI) status, and death.

This result was confirmed, even when adjusted for smoking history and gender and possible confounding factors, regardless of the interval from the last dose received [36]. The authors did not analyze the association between the duration and the number of cycles of ICI and the severity of COVID-19 in their cohort; however, they concluded that such results may be promising for the safety of the continued use of PD-1 blockade during the COVID-19 pandemic.

It is worth noting that the aforementioned studies are distinguished by the sample sizes reported, the cancer populations examined (with annexed small overlapping), and the different endpoints considered.

In order to explore the relationship between COVID-19 outcome and ICI treatment, Szabados et al. retrospectively evaluated 74 patients affected by genitourinary cancers, receiving single-agent PD-1/PD-L1 or combination ICIs during the pandemic period. Only four patients of the considered cohort contracted the infection, two of whom developed symptoms requiring hospitalization and all of whom overcame the infection. Based on these data, the authors concluded by suggesting that the highest COVID-19 death rate associated with systemic therapy should not necessarily be applied to patients receiving ICI [37]. On other hand, due attention must be paid to the lower mortality rate of genitourinary cancers, even at an advanced stage, in comparison with lung cancer, which is considered to be an increased risk condition. This pathological status may be considered as a risk factor of mortality for COVID-19 also due to the difficulty of suspecting SARS-CoV2 infection in lung cancer patients, given the inconsistency of infection-related symptoms, which are hardly distinguishable by those observed in the case of disease progression, superinfection, or treatment-related interstitial pneumonitis.

The case report by Di Noia et al., relating to a 53-year-old man treated with ICI for metastatic non-small cell lung cancer (NSCLC), strengthened this suggestion. Specifically, this cancer patient, long treated with nivolumab (PD-1 inhibitor), who developed a hyper-acute and fatal interstitial pneumonitis, was found to be infected with SARS-CoV-2. The atypical time of onset and the explosive clinical course could be due to an overlap between the underlying disease, the current treatment regimen, and the infection. This negative synergy, combined with a possible diagnostic delay, cannot be excluded or overshadow the safety of immunotherapy during this pandemic [38].

Conversely, Rolfo et al. reported two cases of patients with NSCLC in long treatment with combined therapy, including ICI, who were characterized by a completely different behavior. Both patients, who were diagnosed with SARS-CoV2 infection, presented minimal respiratory symptoms; however, surprisingly, their infection manifested extensive cutaneous involvement. Precisely, the symptoms involved an extensive urticarial reaction and multiform erythema with a targetoid morphology, respectively; although these were initially suggested to be ICI reactions, they turned out to be COVID-19 manifestations [39].

Likewise, O’Kelly et al. reported a positive outcome in a young female affected by a refractory Hodgkin lymphoma and approaching the seventh cycle of Pembrolizumab, which is an ICI targeting PD-L1; she also contracted SARS-CoV2 [40]. During the first six days from COVID-19 diagnosis, she became pyretic, requiring supplementary oxygen due to bilateral pneumonia. Nevertheless, her conditions improved until the thirteenth day, with gradual recovery from the infection. However, it is necessary to underline that this report, apart from being a single case, is bounded by the absence of significant comorbidities associated with an increased risk of hospitalization.

In this regard, Yekeduz et al. reported the clinical course of SARS-CoV-2 infection in a patient with metastatic malignant melanoma, who took nivolumab. This patient, despite their old age, comorbidities, and cancer diagnosis, had no signs of pulmonary involvement either clinically or radiologically [41]. Similarly, the case report presented by Schmidle et al. referred to a patient with metastatic melanoma in the adjuvant immunotherapy with nivolumab, who tested positive for SARS-CoV-2. This patient did not develop any severe respiratory symptoms [42]. In both of the above-mentioned works, the authors go so far as to speculate that the good clinical course of the patients could be related to the blockade of the PD-1/PD-L1 pathway.

Moreover, a multicentric analysis, conducted on 113 heterogeneous oncology patients who tested positive for COVID-19 and were undergoing therapy with ICI, showed that the mortality rate was not higher than that reported for the general oncology population during the pandemic [43].

A recently opened multicenter observational study is currently collecting data in an open consortium called The Thoracic Cancers International COVID-19 Collaboration (TERAVOLT). With the ambition to define the optimal diagnostic and therapeutic practices, this study is aimed at understanding the effect of SARS-CoV2 infection on patients with thoracic malignancies. Regarding systemic treatments, particular attention has been given to ICI therapy due to the conflicting evidence reported in the literature. A preliminary analysis of the available data showed that ICI therapy is not associated with an increased risk of COVID-19 mortality among patients with thoracic cancer [44]. A more recent single-center retrospective analysis examined the proportion of SARS-CoV-2 IgM- and/or IgG-positive subjects in cancer patients either treated with ICIs or undergoing standard chemotherapies and healthcare provisions. A significantly lower percentage of ICI patients were positive when compared with chemotherapy patients. The effect of treatment on seropositivity, corrected by performing propensity score matching based on gender and tumor stage, revealed that ICIs could represent the only protective factor against the onset of COVID-19 infection in cancer patients [45].

However, at a preliminary stage and awaiting more consistent data on the possible immune intersection between COVID-19 and cancer therapy, these findings suggest that postponing or discontinuing such therapies might not be warranted.

## 5. Conclusions

Currently, there is no univocal evidence supporting the interaction between SARS-CoV-2 and ICIs. Nevertheless, based on the limited and unclear data available, a mutual and harmful effect cannot be excluded. Consequently, oncologists are on the horns of a dilemma about how to balance the risk of COVID-19 exposure while delivering effective therapy and assistance. The decision to start, continue, or suspend ICI treatment should be based on case-by-case approaches. Indeed, physicians must consider several variables in this risk–benefit assessment. A temporary discontinuation of treatment with ICI could be reasonable in patients who had long-term control of the disease with maintenance therapy [46]. Likewise, for patients who are starting treatment, the choice between ICI and another treatment option, with the same indication, should be made based on the available efficacy and safety data in that setting of disease.

The European Society for Medical Oncology (ESMO) has given dedicated recommendations for the management of various aspects of oncological care in order to overcome the various clinical and technical areas of uncertainty, ranging from diagnosis to therapeutic planning and the treatment of cancer patients during the COVID-19 outbreak. According to these statements, where there is a significant survival benefit, ICIs should not be withheld or delayed for the approved indications of treatment. In patients who have tested positive for SARS-CoV-2, the (neo) adjuvant ICI should be postponed until recovery [47]. Similarly, the largest Italian organization for cancer research, Alliance Against Cancer (Alleanza Contro il Cancro, ACC), is taking on a scientific leadership in addressing COVID-19 challenges in order to develop “dynamic” indications in fighting such an unpredictable pandemic, including ICI indications and the timing of administration [48]. Nowadays, it is still unclear on which basis some patients have a favorable clinical course while others do not. Can it be related to the time elapsed between the last ICI infusion and SARS-CoV-2 infection? Can it depend on the immunological state of each patient? Indeed, the data are so limited that a hasty conclusion cannot be drawn. Therefore, new studies are required, given the course of the second wave of COVID-19, which seems to have a greater impact than that of the first one.

## Data Availability

No new data were created or analyzed in this study. Data sharing is not applicable to this article.
